# Sex differences in heart failure mortality by ejection fraction subtype: a single-center, retrospective cohort study

**DOI:** 10.3389/fcvm.2025.1686845

**Published:** 2025-12-12

**Authors:** Abiden Kapar, Xingli Gu, Fengwei Liang, Xuanjie Hu, Dandan Tang

**Affiliations:** 1 School of Biomedical Engineering, Hainan University, Sanya, China; 2 College of Medical Engineering and Technology, Xinjiang Medical University, Urumqi, China; 3 Institute of Medical Engineering Interdisciplinary Research, Xinjiang Medical University, Urumqi, China; 4 The First Affiliated Hospital of Xinjiang Medical University, Urumqi, China

**Keywords:** heart failure, left ventricular ejection fraction, all-cause mortality, sex differences, Cox proportional hazards model

## Abstract

**Background:**

Heart failure (HF) is a complex clinical syndrome characterized by structural or functional impairments in ventricular filling or ejection, leading to significant morbidity and mortality. This study investigates sex-specific differences in mortality across HF subtypes: heart failure with reduced ejection fraction (HFrEF), mildly reduced ejection fraction (HFmrEF), and preserved ejection fraction (HFpEF).

**Methods:**

A single-center, retrospective cohort study was conducted at the First Affiliated Hospital of Xinjiang Medical University, China, from August 16, 2012, to January 1, 2025. Patients diagnosed with HF based on the 2018 Chinese Guidelines for the Diagnosis and Treatment of Heart Failure were included. Data on demographics, clinical parameters, and HF subtypes were extracted. Cox proportional hazards regression models assessed all-cause mortality risk, with Kaplan–Meier analysis for survival curves.

**Results:**

Of 12,282 HF patients screened, 6,091 were included (median age: 69 years, IQR: 60–78). Females were older (median age: 72 vs. 67 years, *p* < 0.001) and had higher left ventricular ejection fraction (LVEF) (50.47% ± 11.47% vs. 45.61% ± 10.83%, *p* < 0.001) than males. Hypertension was prevalent in 61.3% of patients. Cox regression identified age as an independent risk factor for all-cause mortality (aHR: 1.04, 95% CI: 1.01–1.06, *p* = 0.002). In the HFmrEF subgroup, female sex was an independent predictor of mortality (aHR: 4.75, 95% CI: 1.47–15.35, *p* = 0.009). Age also predicted mortality in HFpEF (aHR: 1.04, 95% CI: 1.00–1.07, *p* = 0.025). No significant sex-based differences were observed in overall mortality (*p* = 0.12).

**Conclusions:**

Age is a significant independent risk factor for all-cause mortality in HF patients, with female sex emerging as a critical predictor in the HFmrEF subtype. These findings highlight the need for sex-specific risk stratification and tailored management strategies, particularly for females with HFmrEF. Future prospective, multi-center studies are needed to validate these results and explore underlying mechanisms to optimize HF outcomes.

## Introduction

Heart failure (HF) is a complex clinical syndrome resulting from any structural or functional impairment of ventricular filling or ejection, driven by a variety of underlying etiologies. The cardinal manifestations of HF include dyspnea and fatigue—which may limit exercise tolerance—as well as fluid retention that can lead to pulmonary and systemic congestion and peripheral edema (1–3). The clinical syndrome of HF may result from disorders of the pericardium, myocardium, endocardium, heart valves, or great vessels or from certain metabolic abnormalities, but most patients with HF have symptoms due to impaired left ventricular (LV) myocardial function (4, 5). HF has become a growing public health challenge associated with substantial morbidity and mortality (6, 7), placing an unprecedented cost burden on healthcare systems worldwide. Despite widespread adoption of evidence-based pharmacological and device therapies, an unacceptably high number of patients continue to experience functional impairment, poor quality of life, and premature death due to HF (8, 9).

According to the classifications by organizations such as the Heart Failure Society of America, the Heart Failure Association of the European Society of Cardiology, the Japanese Heart Failure Society, and the Writing Committee for the Universal Definition of Heart Failure, HF is categorized as follows: HF with reduced ejection fraction (HFrEF) (HF with LVEF ≤ 40%), HF with mildly reduced ejection fraction (HFmrEF) (HF with LVEF 41%–49%), and HF with preserved ejection fraction (HFpEF) (HF with LVEF ≥ 50%) (10–12).

Notable sex differences exist in cardiac physiology and function, leading to distinct clinical presentations and outcomes (13–15). Typically, females develop HF later in life, primarily due to hypertension, obesity, or diabetes-related microvascular changes associated with HFpEF (14, 16), whereas males are more likely to experience HFrEF secondary to macrovascular ischemia (17). Beyond structural and etiological differences, hormonal variations influence ventricular remodeling in females, resulting in symptom profiles that differ from those in males (18). The purpose of this study is to compare differences between sexes and among various HF subtypes.

## Methods

### Study design and setting

We conducted a single-center, retrospective cohort study at the First Affiliated Hospital of Xinjiang Medical University, Xinjiang Uygur Autonomous Region, China, from August 16, 2012, to January 1, 2025. The study enrolled hospitalized patients with HF, all of whom underwent echocardiography during their initial hospitalization. The diagnosis of HF was strictly based on the criteria outlined in the Chinese Guidelines for the Diagnosis and Treatment of Heart Failure (2018 edition). We included patients hospitalized for HF and extracted comprehensive data from the hospital medical information database. The collected data encompassed: (1) Demographic characteristics: sex, age, occupation, body mass index (BMI), and number of comorbidities; (2) Medical history: family history and cardiovascular family history; (3) Clinical parameters: vital signs, hypertension, coronary disease, left ventricular ejection fraction (LVEF), left ventricular end-diastolic diameter (LVDD), echocardiographic findings, laboratory test results, length of hospitalization, in-hospital outcomes, mortality, and HF subtypes (reduced, mildly reduced, and preserved).

### Study participants and procedure

Patients were categorized into three HF subtypes based on LVEF: HF with reduced ejection fraction: LVEF ≤ 40% (1, 10); HF with mildly reduced ejection fraction: 41% ≤ LVEF ≤ 49%; HF with preserved ejection fraction: LVEF ≥ 50%.

### Participant selection criteria

The study enrolled adults aged 18 years or older who had been diagnosed with heart failure (HF) based on the criteria outlined in the Chinese Guidelines for the Diagnosis and Treatment of Heart Failure (2018) (19). Eligible participants were those classified as New York Heart Association (NYHA) functional class II to IV (20), reflecting moderate to severe heart failure symptoms. Additionally, all participants were required to have received heart failure-specific treatment during their hospitalization to ensure relevance to the study's focus on HF management.

To maintain the study's focus and data integrity, individuals were excluded if their hospitalization was primarily for conditions unrelated to heart failure. Furthermore, participants were not included if critical data, such as left ventricular ejection fraction (LVEF) measurements or hospitalization duration records, were unavailable, as these were essential for the study's analyses. Those with a diagnosis of renal insufficiency were also excluded to avoid confounding factors related to kidney function. Lastly, individuals lacking essential baseline or follow-up information were not included to ensure comprehensive data for analysis.

### Outcome measures and covariates

The primary outcome of this study was all-cause mortality. The primary analysis examined sex-based differences in all-cause mortality, while secondary outcomes assessed mortality differences among HF subtypes (HFrEF, HFmrEF, and HFpEF). Follow-up began at hospital admission and continued until outcome occurrence (in-hospital death).

The analyzed covariates included: sex, occupation, BMI, number of comorbidities, hypertension, coronary disease, family history, cardiovascular family history, LVEF, LVDD, mortality and HF subtypes (reduced, mildly reduced, and preserved).

### Statistical analysis

Continuous variables were presented as mean with interquartile range (IQR), while categorical variables were expressed as counts and percentages. Group comparisons for continuous variables were performed using Student's *t*-test, and chi-square tests were used for categorical variables, with a *p*-value < 0.05 considered statistically significant. Kaplan–Meier (K-M) analysis was employed to plot cumulative mortality curves for all-cause mortality, and Cox proportional hazards regression models were applied to estimate hazard ratios (*HR*) with corresponding 95% confidence intervals (95% *CI*).

All data processing and statistical analyses were conducted using R statistical software (version 4.2.3), with the “survival” and “survminer” packages utilized for Cox regression modeling and K-M curve generation.

## Results

**Figure 1:**
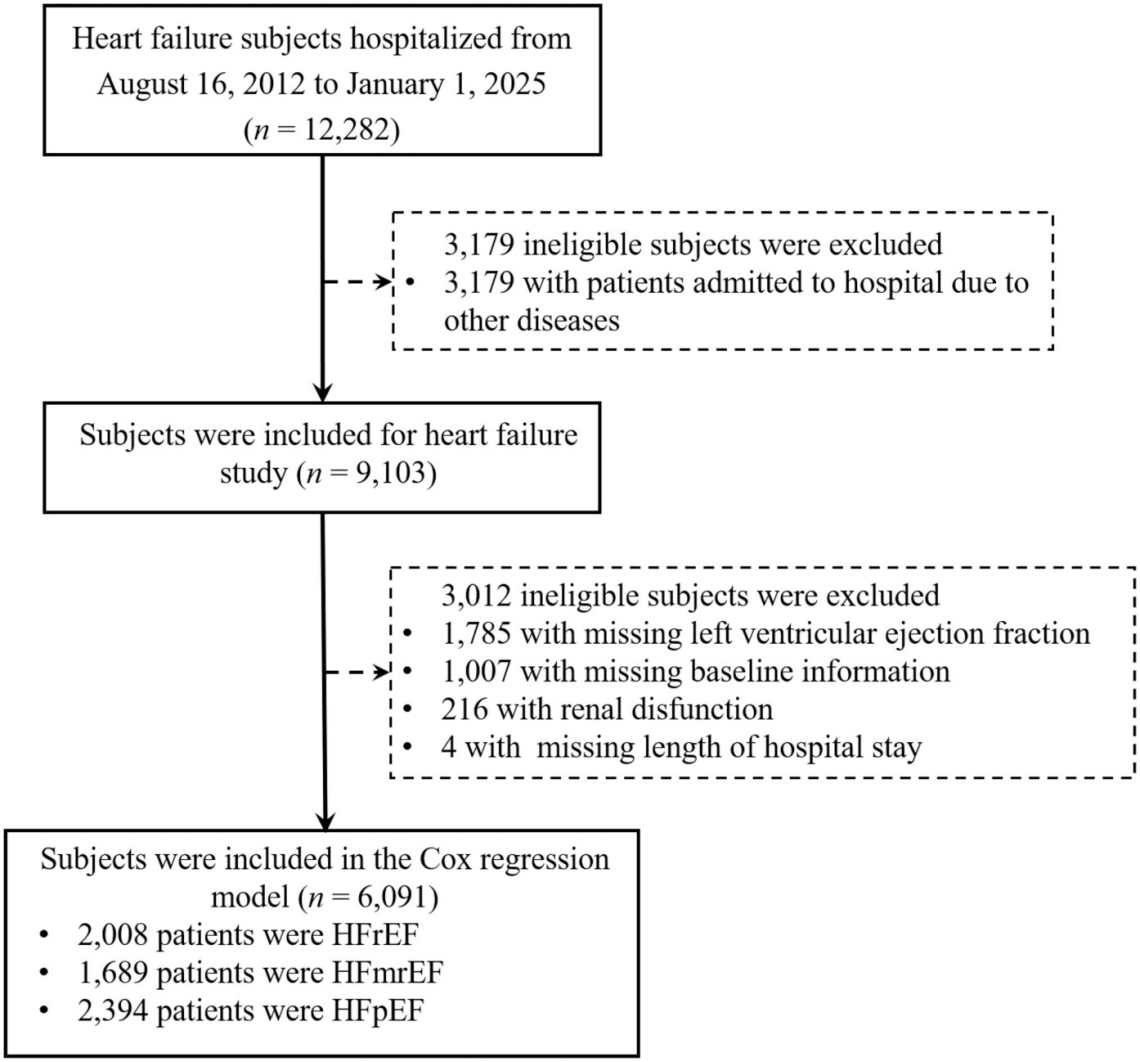
Flowchart of samples selection.

**Figure 2:**
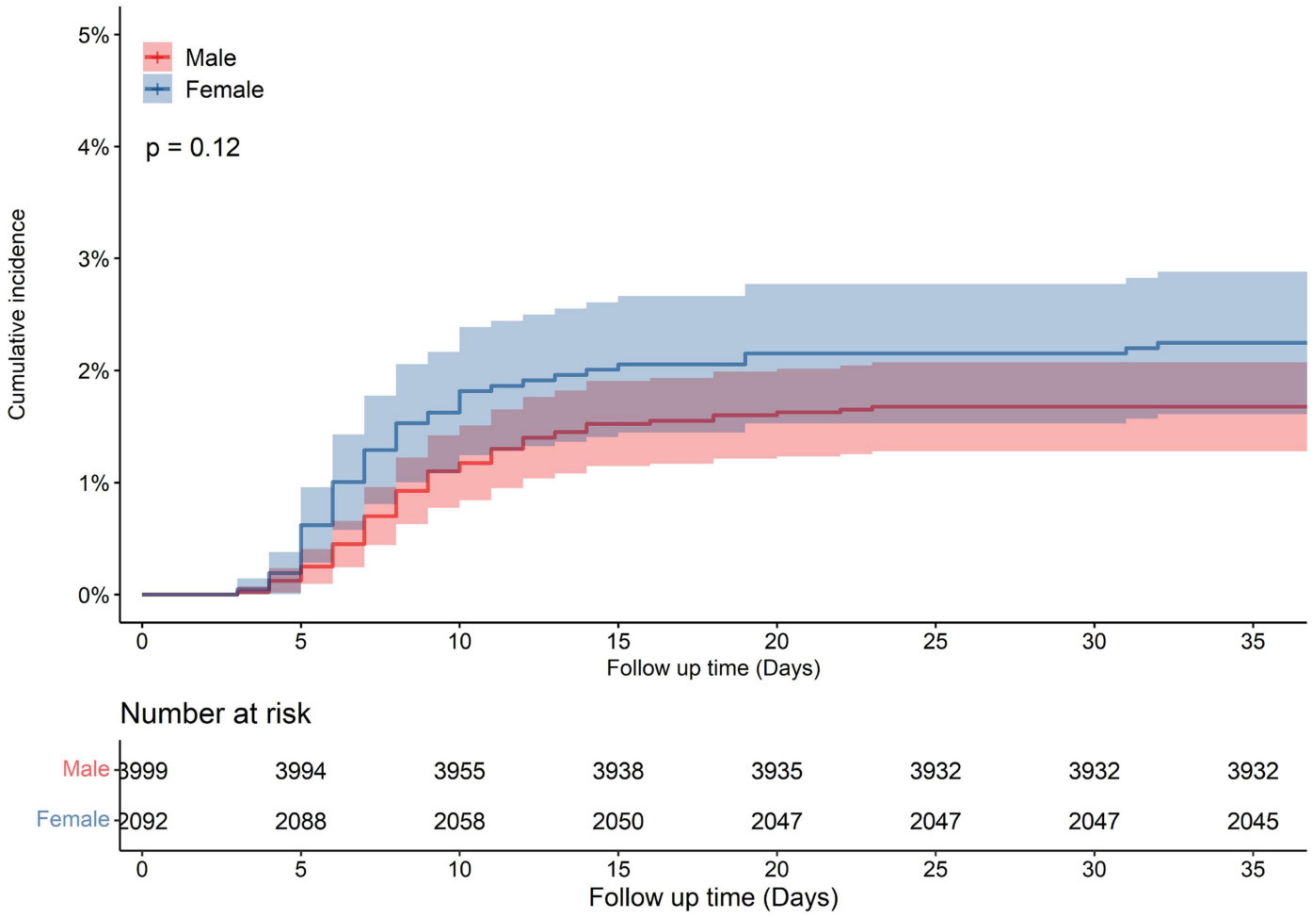
Cumulative incidence in male and female with heart failure patients.

**Figure 3:**
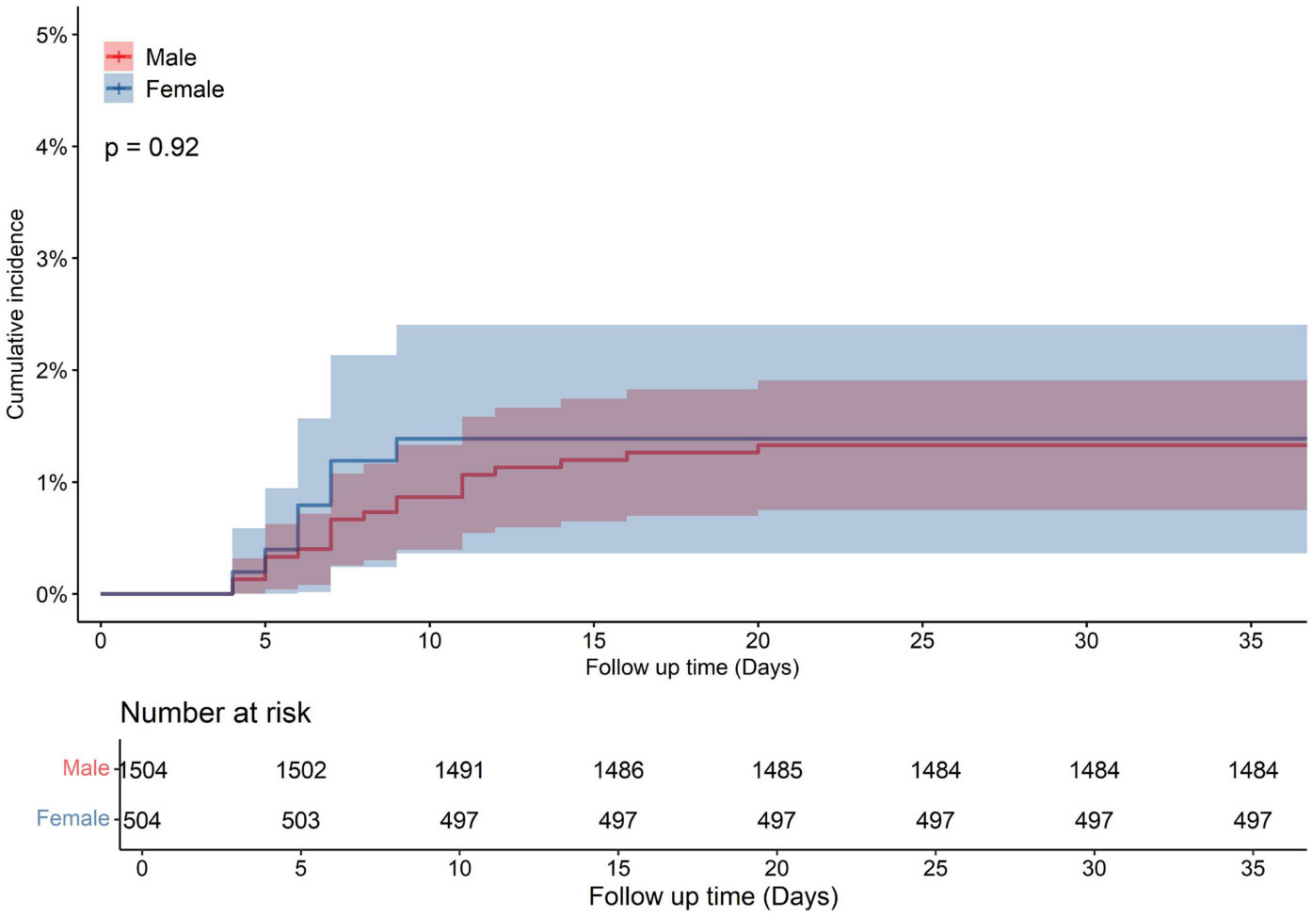
Cumulative incidence in male and female with heart failure reduced ejection fraction (HFrEF) patients.

A total of 12,282 HF patients were initially enrolled. After strict screening, 6,091 patients met the inclusion criteria (Figure 1). The overall population had a median age of 69 years (IQR: 60–78) and a median hospital stay of 6 days (IQR: 5–8). Hypertension was present in 61.3% of patients, and nearly half were retired. Compared to males, female patients were older (median age: 72 vs. 67 years, *p* < 0.001) and had a higher LVEF (50.47% ± 11.47% vs. 45.61% ± 10.83%, *p* < 0.001) (Table 1). All-cause mortality was numerically higher in females than in males (*p* = 0.12, Figure 2). A similar trend was observed in the HFrEF subtype (*p* = 0.92, Figure 3), whereas the opposite pattern was noted in the HFpEF subtype (*p* = 0.81, Figure 5). In terms of HF type distribution, the proportion of males was significantly higher than that of females in both HFrEF and HFmrEF (74.9% vs. 25.1% and 71.3% vs. 28.7%, respectively) (Table 2).

**Table 1:** Sex differences in clinical and echocardiographic characteristics of heart failure patients.

Characteristics	All patients *N* = 6,091	Male *N* = 3,999	Female *N* = 2,092	*p*
Age, year, median (IQR)	69.00 (60.00, 78.00)	67.00 (58.00, 76.00)	72.00 (63.00, 80.00)	<0.001
Occupation, *n* (%)				
Farmers	1,348 (22.1)	829 (20.7)	519 (24.8)	<0.001
Retirees	2,975 (48.8)	1,894 (47.4)	1,081 (51.7)	
Other occupational groups	1,768 (29.0)	1,276 (31.9)	492 (23.5)	
Body mass index group, *n* (%)				
Underweight: < 18.5 kg/m^2^	161 (3.2)	68 (2.0)	93 (5.4)	<0.001
Normal weight: 18.5–24.9 kg/m^2^	2,057 (40.5)	1,287 (38.3)	770 (44.6)	
Overweight: 25–30 kg/m^2^	1,957 (38.5)	1,388 (41.3)	569 (33.0)	
Obesity: > 30 kg/m^2^	909 (17.9)	615 (18.3)	294 (17.0)	
BMI, kg/m^2^, median (IQR)	25.71 (22.98, 28.72)	25.95 (23.42, 28.73)	24.99 (22.04, 28.52)	<0.001
Number of comorbidities, *n* (%)				
*N* = 1	2,125 (34.9)	1,458 (36.5)	667 (31.9)	0.003
*N* = 2	1,693 (27.8)	1,101 (27.5)	592 (28.3)	
*N* = 3	1,045 (17.2)	656 (16.4)	389 (18.6)	
*N* = 4	572 (9.4)	377 (9.4)	195 (9.3)	
*N* ≥ 5	656 (10.8)	407 (10.2)	249 (11.9)	
Hypertension, *n* (%)	3,736 (61.3)	2,454 (61.4)	1,282 (61.3)	0.971
Coronary, *n* (%)	1,302 (21.4)	928 (23.2)	374 (17.9)	<0.001
Diabetes, *n* (%)	1,933 (31.7)	1,293 (32.3)	640 (30.6)	0.175
Family history, *n* (%)	1,599 (26.3)	1,102 (27.6)	497 (23.8)	0.002
Family history of cardiovascular disease, *n* (%)	1,144 (18.8)	777 (19.4)	367 (17.5)	0.079
LVEF, (%), mean (SD)	47.28 (11.29)	45.61 (10.83)	50.47 (11.47)	<0.001
LVDD, mm, mean (SD)	58.18 (9.70)	60.48 (9.46)	53.77 (8.56)	<0.001
HFrEF, *n* (%)	2,008 (33.0)	1,504 (37.6)	504 (24.1)	<0.001
HFmrEF, *n* (%)	1,689 (27.7)	1,205 (30.1)	484 (23.1)	
HFpEF, *n* (%)	2,394 (39.3)	1,290 (32.3)	1,104 (52.8)	
Death, *n* (%)	114 (1.9)	67 (1.7)	47 (2.2)	0.144

**Table 2:** Clinical characteristics of the patients with heart failure with reduced (HFrEF), mildly reduced (HFmrEF), and preserved (HFpEF) left ventricular ejection fraction.

Characteristics	HFrEF *N* = 2,008	HFmrEF *N* = 1,689	HFpEF *N* = 2,394	*P*
Age, year, median (IQR)	65.00 (57.00, 73.00)	68.00 (59.00, 77.00)	73.00 (63.00, 82.00)	<0.001
Sex, *n* (%)				
Male	1,504 (74.9)	1,205 (71.3)	1,290 (53.9)	<0.001
Female	504 (25.1)	484 (28.7)	1,104 (46.1)	
Occupation, *n* (%)				
Farmers	513 (25.5)	369 (21.8)	466 (19.5)	<0.001
Retirees	815 (40.6)	816 (48.3)	1,344 (56.1)	
Other occupational groups	680 (33.9)	504 (29.8)	584 (24.4)	
Body mass index group, *n* (%)				
Underweight: < 18.5 kg/m^2^	45 (2.6)	34 (2.4)	82 (4.3)	0.002
Normal weight: 18.5–24.9 kg/m^2^	690 (40.0)	583 (40.6)	784 (40.7)	
Overweight: 25–30 kg/m^2^	670 (38.9)	590 (41.1)	697 (36.2)	
Obesity: > 30 kg/m^2^	318 (18.5)	229 (15.9)	362 (18.8)	
BMI, kg/m^2^, median (IQR)	25.78 (23.35, 28.73)	25.70 (23.23, 28.65)	25.69 (22.49, 28.89)	0.213
Number of comorbidities, *n* (%)				
*N* = 1	818 (40.7)	606 (35.9)	701 (29.3)	<0.001
*N* = 2	589 (29.3)	468 (27.7)	636 (26.6)	
*N* = 3	304 (15.1)	312 (18.5)	429 (17.9)	
*N* = 4	152 (7.6)	155 (9.2)	265 (11.1)	
*N* ≥ 5	145 (7.2)	148 (8.8)	363 (15.2)	
Hypertension, *n* (%)	1,145 (57.0)	1,055 (62.5)	1,536 (64.2)	<0.001
Coronary, *n* (%)	421 (21.0)	386 (22.9)	495 (20.7)	0.213
Diabetes, *n* (%)	648 (32.3)	550 (32.6)	735 (30.7)	0.371
Family history, *n* (%)	566 (28.2)	450 (26.6)	583 (24.4)	0.014
Family history of cardiovascular disease, *n* (%)	435 (21.7)	305 (18.1)	404 (16.9)	<0.001
LVEF, (%), mean (SD)	34.84 (4.42)	44.68 (2.36)	59.55 (4.34)	<0.001
LVDD, mm, mean (SD)	66.49 (8.16)	59.08 (6.06)	50.58 (6.44)	<0.001
Death, *n* (%)	27 (1.3)	22 (1.3)	65 (2.7)	<0.001

**Figure 4:**
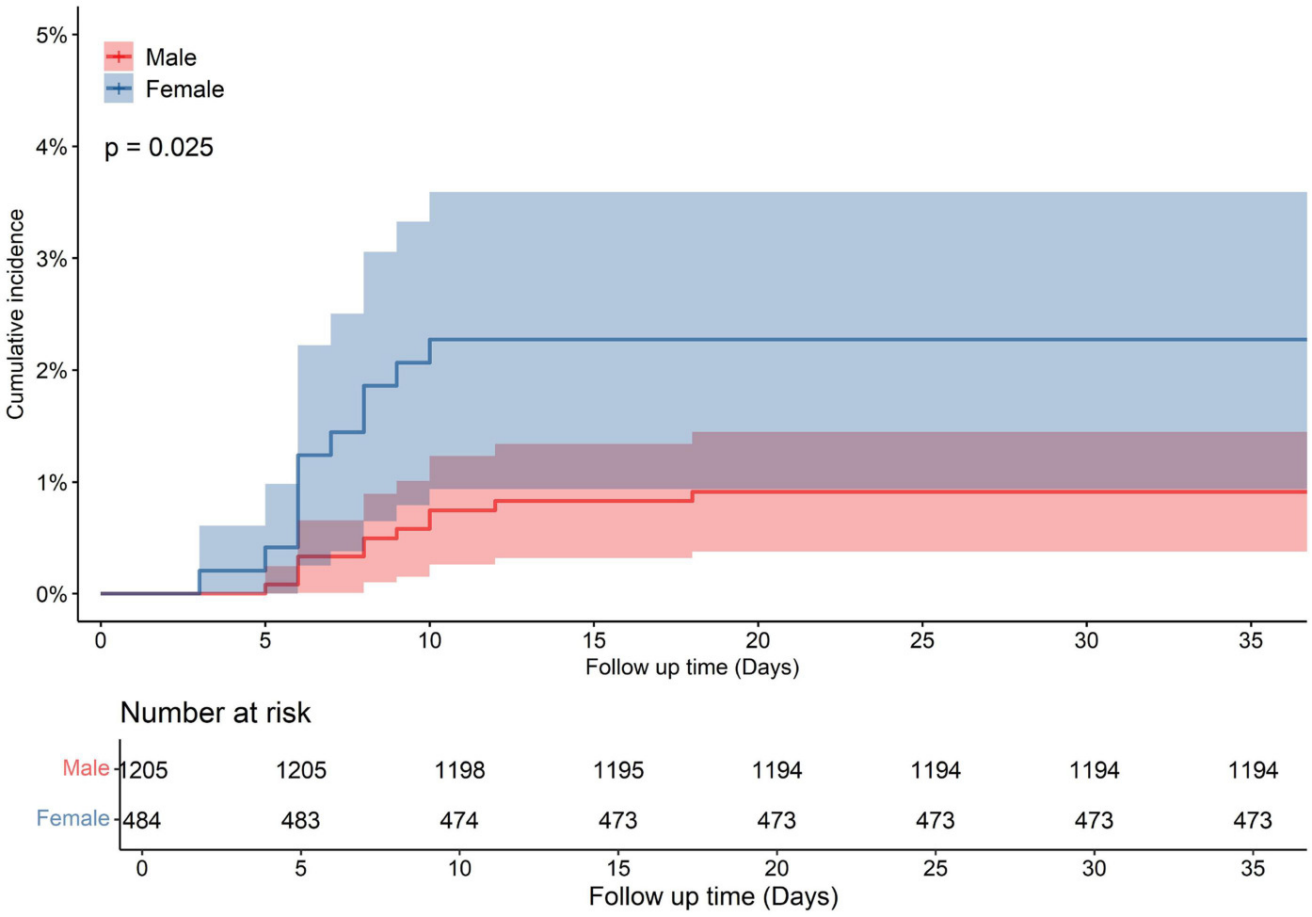
Cumulative incidence in male and female with heart failure mildly reduced ejection fraction (HFmrEF) patients.

Clinical characteristics differed by sex across HF subtypes. Male HFrEF patients had a higher family history disease and larger LVDD. In HFmrEF, male patients had a higher prevalence of coronary disease and larger LVDD, but lower mortality during follow-up compared to females (*p* = 0.025, Figure 4). In HFpEF, male patients had higher rates of coronary disease, higher LVEF, and larger LVDD than females (Table 3).

**Figure 5:**
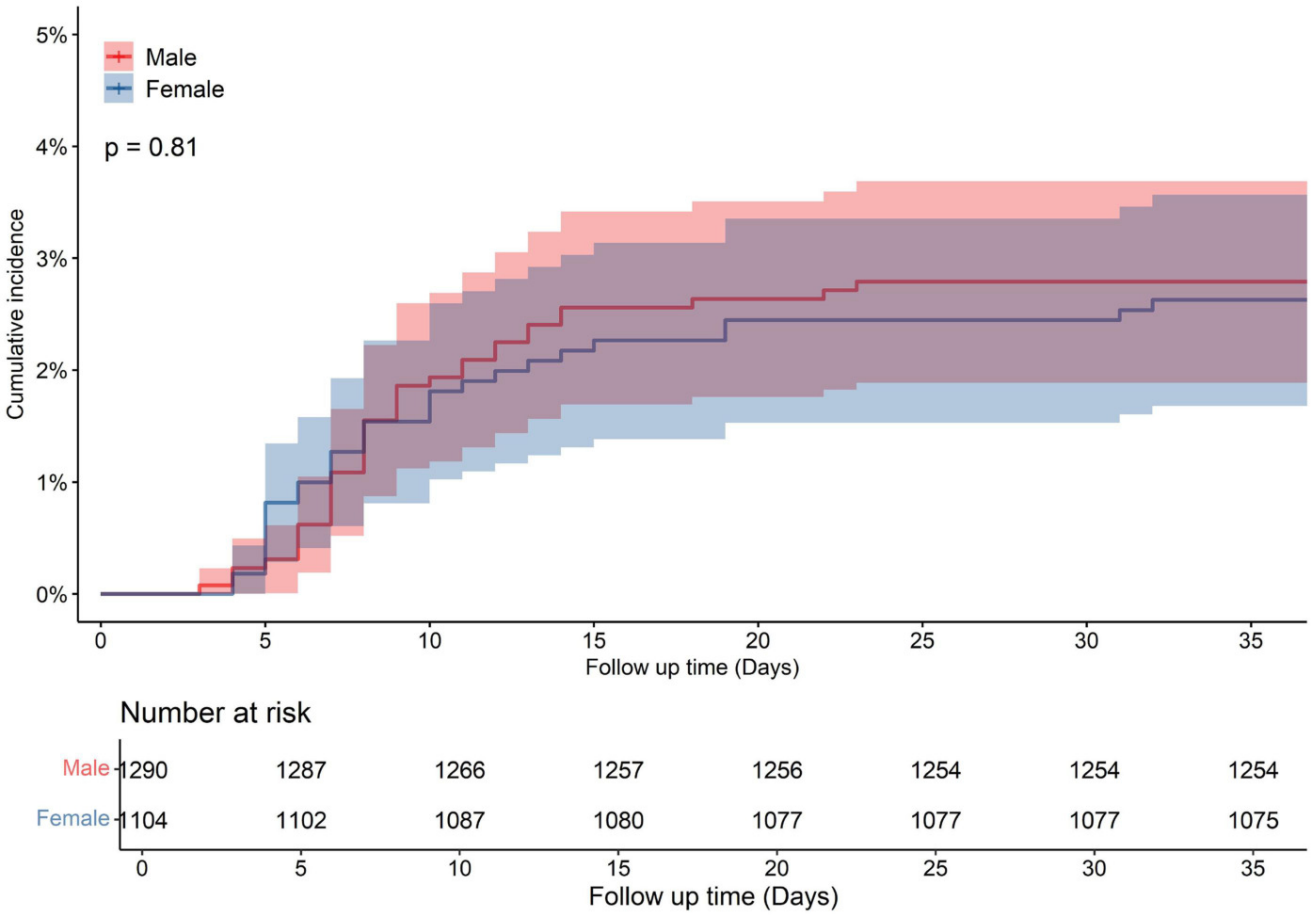
Cumulative incidence in male and female with heart failure preserved ejection fraction (HFpEF) patients.

**Table 3:** Clinical characteristics of sex with heart failure with reduced (HFrEF), mildly reduced (HFmrEF), and preserved (HFpEF) left ventricular ejection fraction.

Characteristics	HFrEF *N* = 2,008		HFmrEF *N* = 1,689		HFpEF *N* = 2,394	
	Male *N* = 1,504	Female *N* = 504	Male *N* = 1,205	Female *N* = 484	Male *N* = 1,290	Female *N* = 1,104
Age, year, median (IQR)	63.00 (55.00, 72.00)	69.00 (60.00, 75.25)*	67.00 (58.00, 76.00)	71.00 (62.00, 78.25)*	71.00 (62.00, 82.00)	75.00 (66.00, 82.00)*
Occupation, *n* (%)						
Farmers	357 (23.7)	156 (31.0)*	241 (20.0)	128 (26.4)*	231 (17.9)	235 (21.3)*
Retirees	609 (40.5)	206 (40.9)*	580 (48.1)	236 (48.8)*	705 (54.7)	639 (57.9)*
Other occupational groups	538 (35.8)	142 (28.2)*	384 (31.9)	120 (24.8)*	354 (27.4)	230 (20.8)*
Body mass index group, *n* (%)						
Underweight: < 18.5 kg/m^2^	19 (1.5)	26 (5.9)*	22 (2.1)	12 (3.0)*	27 (2.6)	55 (6.2)*
Normal weight: 18.5–24.9 kg/m^2^	487 (37.9)	203 (46.2)*	394 (38.2)	189 (46.7)*	406 (38.9)	378 (42.9)*
Overweight: 25–30 kg/m^2^	529 (41.2)	141 (32.1)*	445 (43.2)	145 (35.8)*	414 (39.7)	283 (32.1)*
Obesity: > 30 kg/m^2^	249 (19.4)	69 (15.7)*	170 (16.5)	59 (14.6)*	196 (18.8)	166 (18.8)*
BMI, kg/m^2^, median (IQR)	26.12 (23.53, 29.03)	24.80 (21.90, 27.73)*	25.83 (23.44, 28.72)	25.00 (22.66, 28.30)*	25.95 (22.97, 28.73)	25.25 (21.88, 28.91)*
Number of comorbidities, *n* (%)						
*N* = 1	622 (41.4)	196 (38.9)	444 (36.8)	162 (33.5)	392 (30.4)	309 (28.0)*
*N* = 2	425 (28.3)	164 (32.5)	329 (27.3)	139 (28.7)	347 (26.9)	289 (26.2)*
*N* = 3	233 (15.5)	71 (14.1)	223 (18.5)	89 (18.4)	200 (15.5)	229 (20.7)*
*N* = 4	116 (7.7)	36 (7.1)	105 (8.7)	50 (10.3)	156 (12.1)	109 (9.9)*
*N* ≥ 5	108 (7.2)	37 (7.3)	104 (8.6)	44 (9.1)	195 (15.1)	168 (15.2)*
Hypertension, *n* (%)	874 (58.1)	271 (53.8)	748 (62.1)	307 (63.4)	832 (64.5)	704 (63.8)
Coronary, *n* (%)	331 (22.0)	90 (17.9)	305 (25.3)	81 (16.7)*	292 (22.6)	203 (18.4)*
Diabetes, *n* (%)	490 (32.6)	158 (31.3)	392 (32.5)	158 (32.6)	411 (31.9)	324 (29.3)
Family history, *n* (%)	445 (29.6)	121 (24.0)*	332 (27.6)	118 (24.4)	325 (25.2)	258 (23.4)
Family history of cardiovascular disease, *n* (%)	332 (22.1)	103 (20.4)	219 (18.2)	86 (17.8)	226 (17.5)	178 (16.1)
LVEF, (%), mean (SD)	34.84 (4.40)	34.83 (4.50)	44.66 (2.34)	44.73 (2.42)	59.06 (4.37)	60.12 (4.24)*
LVDD, mm, mean (SD)	67.51 (8.19)	63.44 (7.24)*	60.20 (6.10)	56.29 (4.97)*	52.56 (6.64)	48.26 (5.33)*
Death, *n* (%)	20 (1.3)	7 (1.4)	11 (0.9)	11 (2.3)*	36 (2.8)	29 (2.6)

*p < 0.05: comparisons between male VS female in the same HF subtype.

Cox regression analysis indicated that age was an independent risk factor for all-cause mortality (aHR: 1.04, 95% CI: 1.01–1.06, *p* = 0.002), with each additional year of age associated with a 4% increase in mortality risk. Additionally, patients with two comorbidities showed a trend toward higher all-cause mortality compared to those with only one comorbidity (aHR: 1.76, 95% CI: 1.00–3.11, *p* = 0.051) (Table 4).

**Table 4:** Cox regression analysis for all-cause mortality in heart failure patients.

Characteristics	Sample size	Event (%)	Univariable analysis		Multivariable analysis	
			*HR* (95% *CI*)	*P*	a*HR* (95% *CI*)	*p*
Age	6,091	114 (1.87%)	1.06 (1.04–1.08)	<0.001	1.04 (1.01–1.06)	0.002
Sex, male	3,999	67 (1.68%)	1.00 (Reference)			
Female	2,092	47 (2.25%)	1.35 (0.93–1.96)	0.117	1.00 (0.62–1.62)	0.994
Occupation, farmers	1,348	9 (0.67%)	1.00 (Reference)			
Retirees	2,975	78 (2.62%)	1.80 (1.05–3.06)	0.031	1.64 (0.88–3.06)	0.119
Other occupational groups	1,768	27 (1.53%)	0.46 (0.32–0.65)	<0.001	0.76 (0.49–1.20)	0.240
Body mass index group, underweight	161	4 (2.48%)	1.00 (Reference)			
Normal weight: 18.5–24.9 kg/m^2^	2,057	42 (2.04%)	0.55 (0.25–1.19)	0.127	0.95 (0.42–2.15)	0.904
Overweight: 25–30 kg/m^2^	1,957	24 (1.23%)	1.10 (0.59–2.05)	0.770	1.09 (0.58–2.06)	0.779
Obesity: >30 kg/m^2^	909	11 (1.21%)	1.20 (0.79–1.83)	0.400	1.20 (0.79–1.84)	0.388
Number of comorbidities, *n* = 1	2,125	30 (1.41%)	1.00 (Reference)			
*N* = 2	1,693	26 (1.54%)	2.34 (1.59–3.44)	<0.001	1.76 (1.00–3.11)	0.051
*N* = 3	1,045	16 (1.53%)	1.58 (1.04–2.41)	0.031	1.49 (0.91–2.46)	0.112
*N* = 4	572	12 (2.10%)	1.20 (0.75–1.90)	0.445	1.33 (0.72–2.46)	0.370
*N* ≥ 5	656	30 (4.57%)	0.97 (0.60–1.57)	0.905	1.35 (0.76–2.41)	0.307
Hypertension	3,736	76 (2.03%)	1.26 (0.85–1.86)	0.241	0.96 (0.59–1.56)	0.856
Coronary	1,302	21 (1.61%)	0.83 (0.52–1.33)	0.437	0.70 (0.40–1.22)	0.204
Diabetes	1,933	44 (2.28%)	1.36 (0.93–1.98)	0.111	1.03 (0.63–1.69)	0.895
Family history	1,599	29 (1.81%)	0.96 (0.63–1.46)	0.837	1.05 (0.45–2.46)	0.905
Family history of cardiovascular disease	1,144	22 (1.92%)	1.03 (0.65–1.65)	0.888	1.21 (0.47–3.14)	0.697
LVEF	6,091	114 (1.87%)	1.03 (1.01–1.04)	0.001	0.98 (0.93–1.05)	0.623
LVDD	6,091	114 (1.87%)	0.97 (0.95–0.99)	0.004	1.01 (0.97–1.05)	0.695
LVEF, HFrEF	2,008	27 (1.34%)	1.00 (Reference)			
HFmrEF	1,689	22 (1.30%)	1.65 (1.20–2.27)	0.002	1.66 (0.59–4.70)	0.337
HFpEF	2,394	65 (2.72%)	1.37 (0.93–2.02)	0.111	1.45 (0.90–2.35)	0.131

Subgroup analysis further revealed that among HFmrEF patients, female was an independent risk factor for all-cause mortality (aHR: 4.75, 95% CI: 1.47–15.35, *p* = 0.009). In HFpEF patients, age remained an independent predictor of mortality (aHR: 1.04, 95% CI: 1.00–1.07, *p* = 0.025) (Table 5).

**Table 5:** Cox regression analysis for all-cause mortality by heart failure with reduced (HFrEF), mildly reduced (HFmrEF), and preserved (HFpEF) left ventricular ejection fraction.

Characteristics	Sample size	Event (%)	Univariable analysis		Multivariable analysis	
			*HR* (95% *CI*)	*P*	a*HR* (95% *CI*)	*p*
HFrEF *N* = 2,008						
Age	2,008	27 (1.34%)	1.04 (1.00–0.07)	0.028	1.02 (0.98–1.07)	0.332
Sex, male	1,504	20 (1.33%)	1.00 (Reference)			
Female	504	7 (1.39%)	1.05 (0.44–2.48)	0.916	0.55 (0.17–1.77)	0.315
Occupation, farmers	513	2 (0.39%)	1.00 (Reference)			
Retirees	815	17 (2.09%)	2.19 (0.73–6.55)	0.161	1.69 (0.53–5.45)	0.376
Other occupational groups	680	8 (1.18%)	0.40 (0.19–0.83)	0.015	0.51 (0.21–1.25)	0.141
Body mass index group, underweight	45	2 (4.44%)	1.00 (Reference)			
Normal weight: 18.5–24.9 kg/m^2^	690	9 (1.30%)	0.39 (0.12–1.24)	0.111	0.42 (0.11–1.62)	0.210
Overweight: 25–30 kg/m^2^	670	6 (0.90%)	2.21 (0.82–5.98)	0.117	2.58 (0.91–7.27)	0.074
Obesity: >30 kg/m^2^	318	4 (1.26%)	0.97 (0.44–2.13)	0.935	0.97 (0.43–2.16)	0.933
Number of comorbidities, *n* = 1	818	12 (1.47%)	1.00 (Reference)			
*N* = 2	589	6 (1.02%)	1.62 (0.67–3.88)	0.283	2.23 (0.69–7.25)	0.182
*N* = 3	304	3 (0.99%)	1.96 (0.75–5.11)	0.166	2.14 (0.73–6.26)	0.165
*N* = 4	152	2 (1.32%)	1.04 (0.35–3.04)	0.946	1.30 (0.30–5.61)	0.721
*N* ≥ 5	145	4 (2.76%)	1.02 (0.33–3.13)	0.975	1.74 (0.45–6.69)	0.420
Hypertension	1,145	14 (1.22%)	0.81 (0.38–1.72)	0.581	0.58 (0.23–1.46)	0.244
Coronary	421	3 (0.71%)	0.47 (0.14–1.56)	0.218	0.31 (0.08–1.21)	0.091
Diabetes	648	11 (1.70%)	1.45 (0.67–3.12)	0.343	1.32 (0.51–3.46)	0.568
Family history	566	8 (1.41%)	1.07 (0.47–2.45)	0.869	1.63 (0.35–7.61)	0.537
Family history of cardiovascular disease	435	6 (1.38%)	1.03 (0.42–2.56)	0.941	1.02 (0.20–5.23)	0.980
LVEF	2,008	27 (1.34%)	0.91 (0.85–0.98)	0.015	0.93 (0.84–1.03)	0.151
LVDD	2,008	27 (1.34%)	1.03 (0.99–1.07)	0.201	1.02 (0.96–1.08)	0.489
HFmrEF *N* = 1,689						
Age	1,689	22 (1.30%)	1.07 (1.03–1.11)	0.001	1.05 (0.99–1.12)	0.106
Sex, male	1,205	11 (0.91%)	1.00 (Reference)			
Female	484	11 (2.27%)	2.51 (1.09–5.80)	0.030	4.75 (1.47–15.35)	0.009
Occupation, farmers	369	2 (0.54%)	1.00 (Reference)			
Retirees	816	16 (1.96%)	1.31 (0.39–4.35)	0.658	1.55 (0.30–7.94)	0.601
Other occupational groups	504	4 (0.79%)	0.41 (0.18–0.95)	0.028	0.80 (0.26–2.46)	0.694
Body mass index group, underweight	34	0 (0.00%)	0 (not estimated)		0 (not estimated)	
Normal weight: 18.5–24.9 kg/m^2^	583	8 (1.37%)	0 (not estimated)		0 (not estimated)	
Overweight: 25–30 kg/m^2^	590	7 (1.19%)	0 (not estimated)		0 (not estimated)	
Obesity: > 30 kg/m^2^	229	0 (0.00%)	0 (not estimated)		0 (not estimated)	
Number of comorbidities, *n* = 1	606	4 (0.66%)	1.00 (Reference)			
*N* = 2	468	9 (1.92%)	2.00 (0.69–5.76)	0.199	0 (not estimated)	
*N* = 3	312	3 (0.96%)	1.49 (0.51–4.38)	0.470	0 (not estimated)	
*N* = 4	155	1 (0.65%)	3.38 (0.86–13.30)	0.082	0 (not estimated)	
*N* ≥ 5	148	5 (3.38%)	0.97 (0.27–3.50)	0.958	0 (not estimated)	
Hypertension	1,055	15 (1.42%)	1.29 (0.52–3.16)	0.580	0.55 (0.18–1.70)	0.298
Coronary	386	6 (1.55%)	1.26 (0.49–3.23)	0.624	1.12 (0.31–3.99)	0.863
Diabetes	550	10 (1.82%)	1.74 (0.75–4.02)	0.198	2.86 (0.93–8.80)	0.067
Family history	450	7 (1.56%)	1.28 (0.52–3.15)	0.584	0.07 (0.00–8.41)	0.275
Family history of cardiovascular disease	305	7 (2.30%)	2.13 (0.87–5.21)	0.099	20.09 (0.16–2,556.38)	0.225
LVEF	1,689	22 (1.30%)	0.92 (0.77–1.10)	0.359	0.98 (0.77–1.26)	0.896
LVDD	1,689	22 (1.30%)	1.00 (0.94–1.08)	0.851	1.07 (0.97–1.19)	0.170
HFpEF *N* = 2,394						
Age	2,394	65 (2.72%)	1.06 (1.03–1.08)	<0.001	1.04 (1.00–1.07)	0.025
Sex, male	1,290	36 (2.79%)	1.00 (Reference)			
Female	1,104	29 (2.63%)	0.94 (0.58–1.53)	0.809	0.67 (0.36–1.26)	0.217
Occupation, farmers	466	5 (1.07%)	1.00 (Reference)			
Retirees	1,344	45 (3.35%)	1.85 (0.91–3.79)	0.091	1.49 (0.65–3.42)	0.348
Other occupational groups	584	15 (2.57%)	0.56 (0.35–0.90)	0.017	0.95 (0.53–1.72)	0.872
Body mass index group, underweight	82	2 (2.44%)	1.00 (Reference)			
Normal weight: 18.5–24.9 kg/m^2^	784	25 (3.19%)	0.73 (0.25–2.11)	0.559	1.35 (0.44–4.13)	0.598
Overweight: 25–30 kg/m^2^	697	11 (1.58%)	0.97 (0.41–2.29)	0.939	0.95 (0.40–2.29)	0.915
Obesity: > 30 kg/m^2^	362	7 (1.93%)	1.53 (0.84–2.76)	0.161	1.50 (0.83–2.73)	0.179
Number of comorbidities, *n* = 1	701	14 (2.00%)	1.00 (Reference)			
*N* = 2	636	11 (1.58%)	2.45 (1.47–4.08)	<0.001	1.66 (0.80–3.45)	0.173
*N* = 3	429	10 (2.33%)	1.47 (0.85–2.54)	0.163	1.41 (0.74–2.68)	0.297
*N* = 4	265	9 (3.40%)	0.92 (0.50–1.67)	0.779	0.92 (0.43–2.00)	0.839
*N* ≥ 5	363	21 (5.79%)	1.06 (0.57–1.96)	0.861	1.41 (0.68–2.94)	0.357
Hypertension	1,536	47 (3.06%)	1.47 (0.85–2.53)	0.167	1.48 (0.73–3.02)	0.277
Coronary	495	12 (2.42%)	0.87 (0.46–1.62)	0.653	0.77 (0.37–1.59)	0.486
Diabetes	735	23 (3.13%)	1.24 (0.75–2.06)	0.405	0.64 (0.31–1.31)	0.220
Family history	583	14 (2.40%)	0.85 (0.47–1.53)	0.589	1.17 (0.41–3.35)	0.770
Family history of cardiovascular disease	404	9 (2.23%)	0.79 (0.39–1.59)	0.506	0.78 (0.22–2.81)	0.701
LVEF	2,394	65 (2.72%)	1.05 (0.99–1.11)	0.108	1.02 (0.94–1.10)	0.711
LVDD	2,394	65 (2.72%)	0.96 (0.92–1.00)	0.050	0.96 (0.91–1.03)	0.238

## Discussion

This study identifies a critical sex-specific disparity in HF outcomes, with female sex emerging as an independent predictor of increased mortality risk in the HFmrEF subgroup. This finding, derived from a single-center retrospective cohort study at Xinjiang Medical University First Affiliated Hospital, underscores the importance of sex as a key prognostic factor in HFmrEF, a subtype that bridges HFrEF and HFpEF. By highlighting this novel association, our work challenges the assumption that sex differences in HF outcomes are consistent across all subtypes and provides a clear direction for re-evaluating clinical and research approaches to HFmrEF.

The elevated mortality risk in females with HFmrEF is a significant finding, as HFmrEF remains less well-characterized than HFrEF or HFpEF. Unlike HFrEF, which is more common in males and often linked to macrovascular ischemia, or HFpEF, which is frequently associated with hypertension and microvascular dysfunction in females, HFmrEF represents a heterogeneous group with unique pathophysiological features (17, 21). It is increasingly recognized that this heterogeneity encompasses patients recovering from prior HFrEF, those transitioning toward HFpEF, and individuals with acute decompensation (1, 11). This pathophysiological diversity likely underlies the variable treatment responses and prognostic patterns observed in HFmrEF, and may be a key factor contributing to the distinct sex-specific outcomes we identified in our cohort. Our results suggest that females with HFmrEF may face worse outcomes due to factors such as older age at diagnosis, distinct comorbidity profiles, or sex-specific hormonal influences on ventricular remodeling. This observation aligns with prior studies noting that females tend to develop HF later in life, often with clinical presentations driven by microvascular or metabolic stressors (22). However, our study extends these insights by pinpointing the prognostic significance of female specifically in HFmrEF, a subgroup often overlooked in large-scale cohort studies that dichotomize HF into HFrEF and HFpEF (23).

The clinical implications of this finding are profound. The higher mortality risk in females with HFmrEF necessitates sex-specific risk stratification in clinical practice. Contemporary guidelines, including the 2022 AHA/ACC/HFSA and 2023 ESC focused update, have begun to recommend differential application of therapies across HF subtypes (1, 24). However, the evidence base for treating HFmrEF remains less robust than for other subtypes, and current recommendations provide little guidance on sex-specific management. Our results advocate for tailored approaches for females with HFmrEF (25, 26). Clinicians may need to prioritize aggressive management of comorbidities, such as hypertension or diabetes, which are more prevalent in females and may exacerbate HFmrEF outcomes (22, 27). Furthermore, the elevated risk in this subgroup calls for enhanced monitoring, potentially incorporating advanced imaging to detect subtle ventricular dysfunction or targeted therapies addressing sex-specific pathophysiological mechanisms. For example, exploring interventions that modulate hormonal influences on myocardial function could improve outcomes in females with HFmrEF (28, 29).

Our findings challenge existing assumptions about sex differences in HF. Previous studies, such as Mansur et al. (30), reported no significant sex-based differences in overall HF mortality, with females exhibiting higher LVEF and smaller LVDD compared to males. In contrast, our study highlights a specific vulnerability in females with HFmrEF, suggesting that the intermediate ejection fraction range may confer unique risks not captured in studies focusing solely on HFrEF or HFpEF (23). Additionally, our results diverge from large prospective cohort studies that reported higher lifetime HF risk in males across all ages, potentially due to their exclusion of the HFmrEF category (31). This discrepancy underscores the importance of recognizing HFmrEF as a distinct entity in HF research and clinical guidelines (32, 33).

The role of sex hormones in HF outcomes also warrants further exploration. While age-related declines in estrogen and testosterone are known to modulate ventricular myocyte mechanics, their impact on HFmrEF remains underexplored (34). Experimental evidence suggests that ovarian hormone deficiency may impair cardiomyocyte contractility, an effect potentially reversible with estrogen replacement therapy (28). In contrast, testosterone deficiency in males may contribute to contractile dysfunction in HFrEF but appears less relevant in HFmrEF (35). These findings suggest that hormonal therapies could be a promising avenue for future research, particularly for females with HFmrEF, to mitigate their elevated mortality risk.

For future research, our study highlights the need for prospective studies to validate the sex-specific mortality risk in HFmrEF and investigate underlying mechanisms, such as microvascular dysfunction, inflammation, or genetic factors (36). Including HFmrEF as a distinct category in large-scale studies is critical, as its omission in prior research may have obscured important sex-based differences (17). Additionally, incorporating detailed medication and procedural data in future analyses could clarify how treatment variations influence outcomes in females with HFmrEF. Our findings also inspire research into novel therapeutic strategies, such as hormone modulation or personalized treatment plans, to address sex-specific risks in HF.

This study has several limitations. As a single-center retrospective cohort study, the findings may be subject to selection bias and unmeasured confounders, potentially limiting generalizability. The absence of detailed medication and surgical intervention data may have influenced outcome assessments, as treatment differences could affect mortality risk. Additionally, the use of all-cause mortality as a composite endpoint, encompassing both cardiac and non-cardiac deaths, may introduce interpretation challenges. Despite these limitations, our study provides a compelling case for prioritizing sex-specific approaches in HFmrEF, with the potential to transform clinical practice and improve outcomes for a vulnerable population.

## Conclusion

This study establishes female sex as an independent predictor of mortality in HFmrEF, offering a novel perspective on sex differences in HF outcomes. By emphasizing the unique prognostic significance of sex in this understudied subtype, our findings advocate for sex-specific risk stratification, tailored therapeutic strategies, and targeted research to address the elevated mortality risk in females with HFmrEF. These insights have the potential to reshape clinical practice and guide future investigations to optimize HF management across sexes and subtypes.

## Data Availability

The raw data supporting the conclusions of this article will be made available by the authors, without undue reservation.
